# Whole-Exome Sequencing of Germline Variants in Non-*BRCA* Families with Hereditary Breast Cancer

**DOI:** 10.3390/biomedicines10051004

**Published:** 2022-04-26

**Authors:** Yaxuan Liu, Hafdis T. Helgadottir, Pedram Kharaziha, Jungmin Choi, Francesc López-Giráldez, Shrikant M. Mane, Veronica Höiom, Carl Christofer Juhlin, Catharina Larsson, Svetlana Bajalica-Lagercrantz

**Affiliations:** 1 Department of Oncology-Pathology, Karolinska Institutet, Bioclinicum, Karolinska University Hospital, 17176 Stockholm, Sweden; kharaziha@gmail.com (P.K.); veronica.hoiom@ki.se (V.H.); christofer.juhlin@ki.se (C.C.J.); catharina.larsson@ki.se (C.L.); svetlana.lagercrantz@ki.se (S.B.-L.); 2 Department of Molecular Medicine and Surgery, Karolinska Institutet, 17176 Stockholm, Sweden; hafdis.helgadottir@ki.se; 3 Department of Clinical Genetics, Karolinska University Hospital, 17176 Stockholm, Sweden; 4 Yale Center for Genome Analysis, Yale University, New Haven, CT 06511, USA; jungmin.choi@yale.edu (J.C.); francesc.lopez@yale.edu (F.L.-G.); shrikant.mane@yale.edu (S.M.M.); 5 Department of Biomedical Sciences, Korea University College of Medicine, Seoul 136701, Korea; 6 Department of Pathology and Cancer Diagnostics, Karolinska University Hospital, 17176 Stockholm, Sweden

**Keywords:** hereditary breast cancer, whole-exome sequencing, germline variants, bioinformatics

## Abstract

Breast cancer is the most prevalent malignancy among women worldwide and hereditary breast cancer (HBC) accounts for about 5–10% of the cases. Today, the most recurrent genes known are *BRCA1* and *BRCA2*, accounting for around 25% of familial cases. Although thousands of loss-of-function variants in more than twenty predisposing genes have been found, the majority of familial cases of HBC remain unexplained. The aim of this study was to identify new predisposing genes for HBC in three non-*BRCA* families with autosomal dominant inheritance pattern using whole-exome sequencing and functional prediction tools. No pathogenic variants in known hereditary cancer-related genes could explain the breast cancer susceptibility in these families. Among 2122 exonic variants with maximum minor allele frequency (MMAF) < 0.1%, between 17–35 variants with combined annotation-dependent depletion (CADD) > 20 segregated with disease in the three analyzed families. Selected candidate genes, i.e., *UBASH3A*, *MYH13*, *UTP11L*, and *PAX7*, were further evaluated using protein expression analysis but no alterations of cancer-related pathways were observed. In conclusion, identification of new high-risk cancer genes using whole-exome sequencing has been more challenging than initially anticipated, in spite of selected families with pronounced family history of breast cancer. A combination of low- and intermediate-genetic-risk variants may instead contribute the breast cancer susceptibility in these families.

## 1. Introduction

Breast cancer is the most prevalent malignancy among women worldwide affecting 1.7 million new cases every year [1]. The overall prognosis is relatively good and today’s treatment strategies and possibilities for early detection in screening programs have contributed to a high survival. In general, 10-year survival is close to 90%; however, there are large variations between patients of different disease stages, ranging from close to 100% survival in stage 0 or 1 disease to 20% in a stage 4 disease.

The occurrence of familial forms of breast cancer has long been recognized, and the present notion is that approximately 10% of breast cancer patients have a genetic background predisposing to the disease [2,3]. Typical features of familial breast cancer are early age of onset, bilateral disease, male breast cancer, coincidence of breast cancer in close relatives, and co-occurrence with associated tumors, mainly ovarian cancer. Examples of hereditary tumor syndromes where breast cancer is a component include the hereditary breast and ovarian cancer (HBOC) syndrome [3], Cowden or *PTEN* hamartoma tumor syndrome [4], Li-Fraumeni or heritable *TP53*-realted cancer syndrome [5], Peutz–Jegher syndrome, and the hereditary gastric cancer syndrome [6].

The first identified breast-cancer-predisposing genes *BRCA1* and *BRCA2* are responsible for about 25% of HBOC [7]. In addition to breast and ovarian cancer, *BRCA1* carriers also have an increased risk of developing other tumors such as prostate cancer and pancreatic cancer [8,9,10].

The development of sequencing techniques such as whole-exome sequencing (WES) has contributed to the identification of more than 20 hereditary breast cancer (HBC)-associated genes, including, among others, *TP53*, *PALB2, PTEN*, *ATM*, and *CHEK2*. *ATM* and *CHEK2* are examples of moderate-penetrance genes with lower lifetime risk of developing breast cancer as compared to the high-penetrance genes *BRCA1* and *BRCA2*, and these moderate-penetrance genes account for around 6% of HBC [11]. However, in most families fulfilling the HBC criteria, the specific breast-cancer-predisposing risk gene has not been identified. In these families, the genetic susceptibility to develop breast cancer can instead be due to more commonly occurring low- and intermediate-risk variants.

In Sweden, more than 9000 individuals are diagnosed with breast cancer each year. In an attempt to outline the frequency of breast-cancer-associated risk genes in Sweden, a Swedish *BRCA1/2* Extended Analysis (SWEA) study was conducted during 2012–2017 collecting nearly 4000 families with HBC, which were genetically tested using a 64-gene panel including the known breast-cancer-associated genes. In the current study, we selected three families that were SWEA-negative but had a striking pedigree of HBC for WES analysis, in an attempt to identify new predisposing genes for HBC. Four potential breast-cancer-predisposing genes, i.e., *UBASH3A, MYH13, UTP11L,* and *PAX7*, were selected for further validation through transfection and ectopic expression of wild-type and mutated constructs followed by mass spectrometry profiling and/or Western blot analyses.

## 2. Materials and Methods

### 2.1. Family Selection

Initially, 17 SWEA-negative families with a striking pedigree of HBC were selected. The probands were approached at the Hereditary Unit at the Oncology clinic at Karolinska University Hospital for inclusion in the study for WES analysis. These probands had thus been screened negatively using the 64-gene panel including the known genes associated with hereditary breast cancer: *BRCA1*, *BRCA2*, *CDH1*, *CHEK2*, *PTEN*, *STK11*, *TP53*, *ATM*, *BARD1*, *BRIP1*, *CDKN2A*, *MRE11A*, *NBN*, *PALB2*, *RAD50*, *RAD51C*, *RAD51D*, *ATR*, *BABAM1*, *BAP1*, *BCCIP*, *BLM*, *BRAP*, *BRCC3*, *BRE*, *C17orf70*, *C19orf40*, *CDK4*, *FAM175A*, *FAN1*, *FANCA*, *FANCB*, *FANCC*, *FANCD2*, *FANCE*, *FANCF*, *FANCG*, *FANCI*, *FANCL*, *FANCM*, *HUS1*, *KLLN*, *MDC1*, *RAD1*, *RAD17*, *RAD51*, *RAD51B*, *RAD52*, *RAD54L*, *RAD9A*, *RBBP8*, *RMI1*, *RMI2*, *SDHB*, *SDHD*, *SLX4*, *TOP3A*, *TOPBP1*, *TP53BP1*, *UIMC1*, *XRCC2*, *XRCC3*, *XRCC4*, and *ZNF350* (personal communication with Prof. Åke Borg, Lund University Cancer Center). In 4 out of these 17 families, we were able to sample blood from both healthy and diseased relatives or from at least three individuals with breast cancer. In one family we were not able to confirm the cancer diagnosis from the paternal side; therefore, this family was excluded. Finally, three families were selected for further analysis.

### 2.2. Family Characteristics

**Figure 1:**
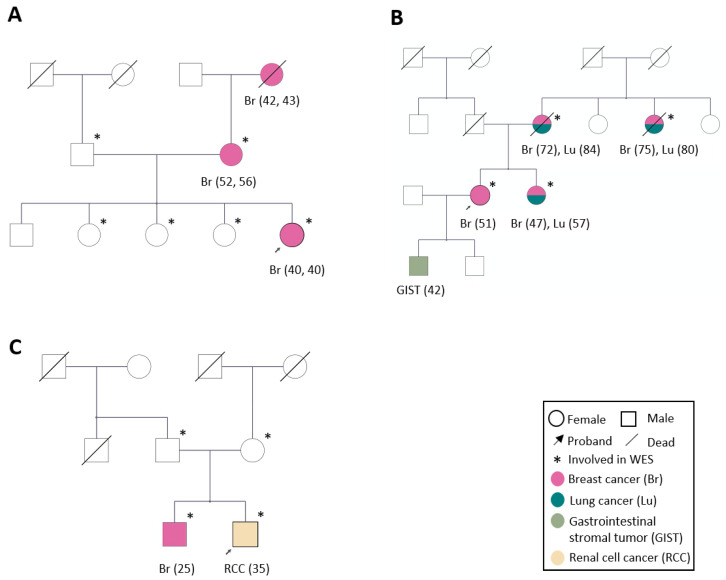
Pedigrees showing the three families in this study. (**A**). Family 1. Three generations with bilateral breast cancer (Br). (**B**). Family 2. Two generations with two primary tumors, i.e., breast and lung cancer (Lu). (**C**). Family 3. Two brothers with early onset cancer, i.e., breast cancer and renal cell carcinoma (RCC), respectively. The proband in each family is indicated with an arrow. Blood samples for WES analysis were collected from relatives marked with a star (*). Pedigree figures were created by the PhenoTips software [12].

Family 1 is characterized by three generations of bilateral ductal breast adenocarcinoma with disease onset between 40 and 56 years of age (Figure 1A; Table S1). The index case was diagnosed with bilateral breast cancer at 40 years of age at the first mammogram examination within the national surveillance program. The three healthy sisters have been enrolled in a surveillance program with yearly breast magnetic resonance imaging (MRI) examinations. During the 6 years follow-up, none of them have been diagnosed with breast cancer and their current age is 50, 59, and 61 years, respectively. Blood samples were obtained from the proband, her mother (breast cancer at 52 years of age and contralateral disease at 56 years of age), father, and from the proband’s three healthy sisters. The maternal grandmother who was diagnosed with breast cancer at age 42 and contralaterally at 43 had earlier succumbed to the disease. No tumor material was available despite intensive search at the pathology unit at Karolinska University Hospital.

Family 2 was selected due to four first-degree relatives with breast cancer (age of onset between 47 and 75) of which three were diagnosed with lung cancer (age of onset 57–84), following their breast cancer. All three were non-smokers. The proband was diagnosed exclusively with breast cancer at 51 years of age (no lung cancer) and one of her sons was diagnosed with a gastrointestinal stromal cell tumor (GIST) at the age of 42. Blood samples from all women with breast cancer were collected (Figure 1B; Table S1).

For Family 3, two brothers with early-onset cancer; one with breast cancer at 25 and the other with clear cell renal cell carcinoma at 35. These two brothers and their healthy parents were enrolled in the study (Figure 1C and Table S1).

### 2.3. Whole-Exome Sequencing and Bioinformatics

WES was performed in collaboration with the Yale School of Medicine and the Clinical Genomics, SciLifeLab, Karolinska Institutet. Briefly, genomic DNA was isolated from the blood using Blood & Cell Culture DNA Midi Kit (#13343, Qiagen, Hilden, Germany) and captured using SeqCap EZ version 2 by NimbleGen (Roche NimbleGen, Madison, WI, USA). Captured fragments were sequenced using 76 bp paired-end sequencing reads in an Illumina HiSeq 2000 (Illumina Inc., San Diego, CA, USA) according to Illumina protocols. Sequencing reads were independently aligned to human genome build 37 (GRCh37/hg19) with BWA-MEM [13] and further processed using the GATK 3 Best Practices workflow [14]. Variants were annotated with ANNOVAR [15]. The data was filtered by the max minor allele frequency (MMAF) obtained from the SweGen [16], and GnomAD [17] databases where variants with MMAF ≥ 0.1% were excluded. Furthermore, we selected for exonic variants, as well as for variants segregating with the disease in each family. The functional prediction of the variant was evaluated by combined annotation dependent depletion (CADD) [18], with higher scores (CADD > 20) being more deleterious, and ClinVar [19].

### 2.4. Cell Culture, Transfection, and Plasmid Construction 

The breast cancer cell lines MCF-7 (#HTB-22™, ATCC, Manassas, VA, USA) and MDA-MB-231 (#CRM-HTB-26, ATCC) were cultured in DMEM medium (#31885-023, Gibco, Waltham, MA, USA) with 10% fetal bovine serum, and 3∗10^5^ cells were seeded per well in a 6-well plate 24 h before transfection. After reaching 70–80% cell confluence, the cells were transfected with 2 ug of plasmid per well using lipofectamine 2000 (#11668-019, Invitrogen, Waltham, MA, USA) or lipofectamine 3000 (#L3000-008, Invitrogen) according to the manufacturer’s protocol. Plasmids containing wild-type *UBASH3A*, *MYH13*, *UTP11L*, and *PAX7* in the expression vector pcDNA3.1(+) were purchased from Genscipt Biotech (Piscataway, NJ, USA). The Quickchange II XL Site-directed Mutagenesis Kit (#200521, Agilent Technologies, Santa Clara, CA, USA) was used to construct the corresponding mutant plasmid. These plasmids were validated by Sanger sequencing performed at the KI Gene core facility.

### 2.5. Western Blot

Proteins were isolated from MDA-MB-231/MCF-7 cells transfected with control/wild-type/mutant plasmid and were quantified by BSA assay and 30 ug per sample was prepared with LDS sample buffer and sample reducing agent was heated at 75 °C 10 min and run on a 4–12% Tris-Bis gel with MOPS buffer at 200 V for 1 h. The protein transfer from gel to PVDF membrane with transfer buffer was performed at 40 V for 80 min. Membranes were blocked with 5% no-fat milk in TBST for 1 h and were then incubated overnight with primary antibody at 4 °C. The following day, the membranes were incubated with appropriate secondary antibody for 1 h at 37 °C, followed by developing and analysis on an iBright Western blot imaging system. Primary antibodies used targeted UBASH3A (#CF809272, mouse, Origene, Rockville, MD, USA), MYH13 (#PA5-70713, rabbit, Thermo Fisher, Waltham, MA, USA), UTP11L (#MA5-27022, rabbit, Thermo Fisher), PAX7 (#CF811661, mouse, Origene), EGFR (#4267, rabbit, CST, Danvers, MA, USA), p-EGFR (#3777, rabbit, CST), AKT (#4691, rabbit, CST), p-AKT (#4060, rabbit, CST), ERK (#9102, rabbit, CST), p-ERK (#4376, rabbit, CST), BAX (#14796, rabbit, CST), STAT3 (#12640, rabbit, CST), p-STAT3 (#9145, rabbit, CST), Beta-catenin (#9562, rabbit, CST), and GAPDH (#5174, rabbit, CST). The secondary antibodies used were anti-rabbit (#7074, CST) and anti-mouse (#7076, CST).

### 2.6. Mass Spectrometry and Pathway Analysis

Proteins were harvested from MCF-7 cells transfected with wild-type plasmid or mutant plasmid of *MYH13* and *UTP11* genes, respectively, and mass spectrometry was performed at the Clinical proteomics unit, Karolinska Institutet. Differentially expressed proteins, between the cells transfected with wild-type or mutant plasmid, were imported to the WEB-based Gene Set Analysis Toolkit (http://www.webgestalt.org, accessed on 1 January 2021) [20] for the Kyoto Encyclopedia of Genes and Genomes (KEGG) pathway analysis.

## 3. Results

### 3.1. Hereditary-Breast-Cancer-Associated Genes and Breast-Cancer-Associated High-Risk SNPs

Even though these families were screened negatively using the 64-gene panel including the known genes associated with hereditary breast cancer, we searched for variants in these 64 genes in the generated WES data. Fifty-seven variants in 29 genes with MMAF  <  0.2 were observed, but none of the variants could explain the breast cancer susceptibility observed in these families. Four variants in three genes (*FANCA*, *FANCM*, and *TOPBP1*) were found in patients of Family 1, and a *BRCA2* missense variant (rs80358899) was observed in the proband from Family 2 (Table S2). However, all of them are considered as benign according to ClinVar.

When searching for rare variants contributing to tumorigenesis, we may exclude some more common single-nucleotide polymorphisms (SNPs). In the 24 known breast cancer risk SNPs [21], rs1045485 in CASP8 was only found in Family 2 and was shared by the proband and the proband’s sister (Table S2).

### 3.2. Identification of Candidate Genes by WES Analysis

In total, 93,347 variants were found in all individuals involved in this WES study, including exonic and exon–intron boundary variants. None of the variants were shared by the healthy or affected individuals when comparing the three families.

**Figure 2:**
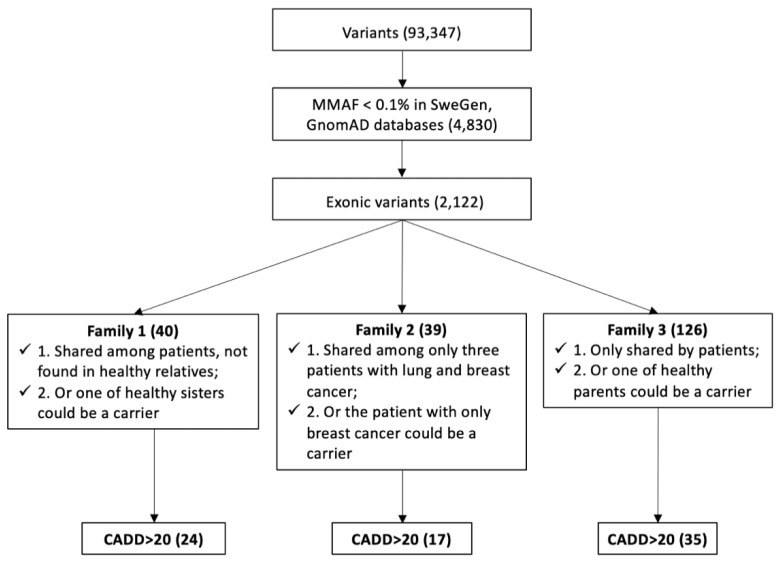
The workflow of variant selection in three families. Variants were filtered by max minor allele frequency (MMAF), exonic location, family segregation and combined annotation dependent depletion (CADD) scores. For all families, we applied two filter strategies considering the incomplete penetrance. In total, 24, 17, and 35 candidate variants were identified in Family 1, Family 2, and Family 3, respectively.

After filtering of variants with MMAF ≥ 0.1%, 4830 variants remained and after filtering non-exonic variants, 2122 variants were left. These 2122 exonic variants (27 nonsense, 204 splicing, and 1891 missense, Table S3) were selected for further analysis. In the first two families, maternal inheritance of breast cancer was seen. The flow chart of variant selection is summarized in Figure 2.

#### 3.2.1. Candidate Genes in Family 1

To identify new breast-cancer-predisposing genes in Family 1, two different filtering of the data were applied. First, we applied strict filtering where only the affected individuals were considered as carriers of high-risk predisposing variants, and secondly, less strict filtering was used where one healthy sister could also be a carrier.

Firstly, we postulated that the father and the three healthy sisters do not share the risk variant with their affected sisters and mother. Therefore, variants detected in the father or any of the three healthy sisters were excluded, resulting in seven candidate missense variants (CADD > 20, MMAF < 0.001) that could contribute to the disease in the affected sisters (Table 1). Three of the gene variants, *UBASH3A*-rs201756769, *MYH13*-rs767313943, and *FBXL4*-rs757154231, had CADD  >  25 and were considered as high-risk gene variants. The MMAF of rs369992593 in the *STRADB* gene, rs141755850 in the *CLK1* gene, *FBXL4,* and *MYH13* missense variants were less than 0.0001.

The *UBASH3A* and *MYH13* variants were functionally assessed in MDA-MB-231 and MCF-7 breast cancer cell lines. Concerning *UBASH3A*, the wild-type UBASH3A protein was highly elevated while the mutated protein showed only a slight increase compared to the basal expression of UBASH3A in both cell lines after transfection (data not shown). The difference between the biological effect of wild-type protein and mutated protein was therefore hard to clarify. After ectopic expression of the MYH13 (Figure S1A), no differences were observed between the mutant and wild-type MYH13 in terms of expressed proteins involved in pathways of proliferation, apoptosis, or adhesion by Western blot (Table S4). We identified 719 differentially expressed proteins from mass spectrometry using MCF-7, and a KEGG pathway analysis revealed that these proteins were enriched for seven different pathways, of which “DNA replication” and “protein processing in endoplasmic reticulum” showed statistical significance (adjusted *p* value ≤ 0.05; Figure S1B). However, no cancer-associated proteins in these pathways were found for further analysis.

Secondly, the analysis was performed with the assumption that one of the healthy sisters could be a carrier of the risk variant (non-penetrant), so the selection was performed with removal of variants identified in two of the sisters (in different combinations) instead of all three healthy sisters. With this filtering, we identified additional 16 missense variants and 1 splice variant (CADD > 20, MMAF < 0.001) that segregated with breast cancer (Table 1). The *SPTLC3*-rs372930777 variant had the highest CADD score of 35. The *CAPN2*-chr1:223947063A/G, *COL17A1*-rs757388768, and *HCLS1*-rs757006680 missense variants had the same CADD score of 34. Nine genes were found to have variants with MMAF  <  0.0001 (*SPTLC3*-rs372930777, *CAPN2*-chr1:223947063A/G, *HCLS1*-rs757006680, *CNST*-rs766380272, *APOB*-rs778274241, *TENM4*-rs747100917, *DNAH3*-rs376279103, *NMRK2*-chr19:3933697T/G, and *DLL3*-chr19:39996056G/A; Table 1).

#### 3.2.2. Candidate Genes in Family 2

Two distinct data filtering processes were also applied in Family 2. A rigid filter was used at first, assuming that only patients with lung cancer and breast cancer were considered as carriers of a pathogenic variant in a high-risk susceptibility gene. In the second filtering, we speculated that the proband with breast cancer only also carry the disease-causing variant.

We first identified nine missense variants (CADD > 20, MMAF < 0.001) shared by the three patients with breast and lung cancer, not including the patient with only breast cancer (Table 2). Variants with CADD  >  25 were considered as high-risk variants (*AKR1B1*-rs201718247, *SLC25A25*-rs748220703, *RYR3*-rs760906719, *C1orf228*-rs575641425, *RUFY1*-rs754852607, *HECTD4*-rs779868916, and *TIPIN*-rs200514985). The MMAFs of *RUFY1* and *HECTD4* missense variants were less than 0.0001.

We found eight additional variants (CADD > 20, MMAF < 0.001) that were shared among all four affected family members (Table 2), assuming that the patient with breast cancer only could also be a carrier of the risk variant and possibly later develop lung cancer. There were seven missense variants and one splice-site acceptor variant. rs771377582 in the *UTP11L* gene and rs369607271 in the *PAX7* gene had both high CADD scores of 35 and 34, respectively. Variants with MMAF < 0.0001 were found in three genes (*PAX7*-rs369607271, *ZMYM4*-chr1:35836075G/A, and *WDFY4*-rs748753983).

The variants in *UTP11L* and *PAX7* were further studied. After ectopic expression of UTP11L (Figure S2A) and PAX7 (not shown), respectively, no difference in expression of proteins involved in proliferation, apoptosis, or adhesion pathways was observed between cells transfected with wild-type plasmid or mutated plasmid (Table S4). Concerning *UTP11*, there are limited publications on its involvement in cancer. Therefore, we proceeded with mass spectrometry of MCF-7 and identified 775 differentially expressed proteins that were subjected to KEGG analysis, which did not identify any significantly enriched pathways after false discovery rate adjustment (Figure S2B).

#### 3.2.3. Candidate Genes in Family 3

We applied two strategies to find high-risk susceptibility variants in Family 3 which includes two brothers with early-onset cancer (one with breast cancer and one with renal cell carcinoma) and two healthy parents. Firstly, we assumed that both of the affected individuals shared the same variant, but not their healthy parents. Secondly, we hypothesized that one of parents could be a disease-free carrier (i.e., low penetrance).

Only one variant, chr14:30108088A/C in *PRKD1* (CADD > 20, MMAF < 0.001), was shared by the two brothers (Table 3); however, it has been classified as likely benign in the Clinvar database. Considering the chance that one of the parents could carry the same variant, we found 34 additional variants (CADD > 20, MMAF < 0.001) (Table 3). Of these, *TNRC6C*-rs367710467, *STAB1*-chr3:52554136G/C, and *THOP1*-chr19:2799789G/A had the highest CADD score of 33. There were 31 variants with MMAF < 0.0001 (Table 3).

## 4. Discussion

The aim of this study was to identify genetic variants predisposing for hereditary breast cancer using whole-exome sequencing in families where extended genetic screening within the clinical platform had failed to detect cancer-predisposing genes. We selected three families with a remarkable family history of bilateral breast cancer, breast and lung cancer, and early-onset male breast cancer, respectively, and were able to collect blood samples for genetic analyses from family members covering at least two generations.

The linkage analysis of large pedigrees was the main method in early studies on hereditary cancer to find prevalent high-penetrance cancer risk genes, e.g., *BRCA1* and *BRCA2* associated with breast and ovarian cancer, and *MSH2* and *MLH1* associated with Lynch syndrome [22,23,24,25,26]. The other strategy relies on WES analysis to identify candidate genes in patients with clinical features of hereditary cancer (i.e., early tumor onset, family history, multiple tumors, etc.). However, the impact of WES in revealing the genetic cause in hereditary cancer syndromes has been lower than initially anticipated [27], indicating the challenge with this approach. The use of genome-wide association studies (GWAS) in breast cancer families has allowed the identification of genes that are characterized by incomplete penetrance, e.g., *CHEK2* and *ATM* [28,29]. Furthermore, genes known to be altered in sporadic tumors have also been sought as another source of candidate genes for hereditary cancer forms.

After performing WES analysis in our families with striking breast cancer pedigrees, pathogenic variants in known hereditary-breast-cancer-syndrome-predisposing genes were excluded. However, rs1045485 in *CASP8* was identified in Family 2 as a potential breast cancer risk variant and it was only shared by the proband and the proband’s sister that developed breast cancer at age 51 and 47, respectively. This SNP could potentially explain the earlier onset of breast cancer compared to their mother and aunt that developed breast cancer at age 72 and 75, respectively. In addition, we also included the splicing variants outside the non-canonical splice sites (−/+ 1, 2 bases in the introns) using VarSEAK (www.varSEAK.bio, accessed on 1 January 2021); however, no variants were predicted as likely pathogenic or pathogenic.

To search for new rare variants, the WES and bioinformatic filtering in our three families with HBC revealed 2122 variants that were selected for further analysis. In Family 1, we performed the first filtering by removing variants that were shared by the proband´s father and her three healthy sisters resulting in seven variants of interest. No functional effects were observed in the protein expression analysis of the *UBASH3A* and *MYH13* missense variants, so other variants could be taken into consideration. The *FBXL4* gene is identified as a potential tumor suppressor in prostate cancer, since the loss of *FBXL4* has been correlated with advanced tumor stage and poor survival [30]. Meanwhile, the observed *FBXL4* missense variant is located in the AMN1 domain, which refers to the regulation of MEN pathway in mitosis. The MMAF of the missense variant in the *STRADB* gene was less than 0.0001. The *STRADB* gene plays a role in the cell cycle and an intronic variant in this gene has been associated with ER-positive breast cancer [31]. Among the other interesting candidates, the *CLK1* gene is highly expressed in normal breast tissue, according to the human protein atlas, and has also been linked to the regulation of mRNA-splicing processes in gastric cancer [32]. The *CLK2* gene has been identified as an oncogene in sporadic breast cancer [33]. *CLK1* may have similar function, but no evidence showed its relation to breast cancer. Frameshift variants of the *TAF1C* gene lead to alterations of RNA polymerase I transcription in gastric and colorectal cancers [34]. The *FRZB* gene regulates Wnt signaling, and downregulation of the FRZB protein has been observed in breast cancer [35]. Bernascone et al. [36] found that the loss of FRZB predisposed mice to breast cancer. The observed *FRZB* missense variant is located in the NTR domain (647–976), which may alter the binding to matrix metalloproteinases and possibly affect the downstream Wnt signaling.

It is important to note that this first filtering assumes full disease penetrance, which is not the case in most hereditary cancer syndromes, and we thus risk selecting out disease-causing variants. Considering this possibility of incomplete penetrance, the selection was further performed by removing variants that were identified in at least two healthy sisters instead of all three and then 17 variants remained. One variant with MMAF < 0.0001 and a very high CADD score (34) was located in the *CAPN2* gene. The high expression of CAPN2 has previously been reported as associated with adverse prognosis in triple-negative breast cancer [37]. Knockdown of *CAPN2* mRNA expression reduced breast cancer cell invasion by regulating invadopodia dynamics [38], that is associated with metastasis. The observed variant is located in the Calpain III domain associated with cytoskeletal remodeling processes and cell differentiation [39]. The other variant with a very high CADD score was seen in the *COL17A1* gene, which inhibits cell migration and invasion and is a favorable prognostic marker in breast cancer [40]. The observed variant is located in the Collagen domain involved in the formation of connective tissue structure. These genes could have the potential to increase the risk of breast cancer.

For Family 2, we also performed two different filtering approaches. At first, nine missense variants shared by three patients with breast and lung cancer were selected. Variants with MMAF  <  0.0001 and CADD > 25 were noted in two genes (*RUFY1* and *HECTD4*)*. RUFY1* has been reported to promote disease progression in gastric cancer and *HECTD4* was a favorable prognostic marker in head and neck cancer according to the Human Protein Atlas database. Other interesting gene variants include one in the *AKR1B1* gene. The *AKR1B1* gene can promote breast cancer development by activating the epithelial-to-mesenchymal transition (EMT) process and the observed missense variant is located in its functional domain [41]. Another variant was found in *BCL6* that is reported to contribute to the development of breast cancer. The overexpression of *BCL6* in the breast cell line inhibited the expression of β-casein protein and apoptosis [42]. Moreover, the variant discovered in our study is located in a conserved domain. However, the patient with breast cancer has the potential to develop lung cancer in later years, so we also selected eight variants shared among all four patients. No functional impact of *UTP11L* and *PAX7* variants with high CADD scores was found in following protein expression analyses. The *FNDC3B* gene is an oncogene in hepatocellular cancer and the high expression of *FNDC3B* induces the EMT process, PI3K/Akt, Rb1, and TGFβ signaling [43]. Except for *UTP11L* and *PAX7*, these genes could be candidate genes for breast cancer.

The third family (Family 3) was characterized by two early-onset cancers in two brothers, one with breast cancer and the other with renal cell cancer, while both parents were healthy. Male breast cancer is extremely rare especially at that age [44]. For the first filtering, only one variant with a CADD score above 20 was identified. To find a putative disease-causing gene, we also performed the second filtering as we speculated that the father or mother may carry the variant as well. The *SATB1* gene can contribute to tumor growth in a mammary adenocarcinoma mouse model [45], while *ZNF507* was reported to affect TGF-β signaling to promote prostate cancer [46]. Pathogenic variants in the *DICER1* gene cause the DICER1 syndrome, including benign or malignant tumors of the thyroid, kidney, ovary, brain, and lung [47], but the variant chr14:95570153T/C discovered in our study is not located in a conserved domain; therefore, it may not affect the function of protein. These genes could have the potential to increase the risk of breast cancer. We have searched for a recessive inheritance (i.e., parents with heterozygous variants and brothers with homozygous variants); however, no variants were found. There is of course also a possibility that the brothers developed sporadic tumors with no genetic predisposition.

A limitation of our study is the small cohort of hereditary breast cancer families. The challenge has been to identify families with a striking pedigree of HBC and include only families in whom we were able to draw blood from several generations, and preferably from both healthy and diseased individuals, which leads to a limitation of the cohort size. Another restriction was that we performed WES that focuses on exonic variants, but will miss variants within the intronic areas and may affect splicing. These cannot be identified without a whole-genome-sequencing approach. Moreover, multiple variants in various genes may act in concert and contribute to the disease phenotype, which would also not be picked up by our functional approach testing one variant at the time.

In conclusion, whole-exome sequencing is a useful tool and has led to the identification of many genes associated with rare cancer syndromes. However, it has been more challenging than initially anticipated. In spite of selected families with pronounced family history of breast cancer, we were not able to identify any disease-causing genes with certainty. A wider combination of low- and intermediate-genetic-risk variants may instead contribute the breast cancer susceptibility in these families.

## Figures and Tables

**Table 1:** The twenty-four variants with MMAF < 0.001, CADD > 20 in Family 1.

Filter	Gene	Position	Ref/Alt	SNP	Type	Change	MMAF	CADD
1	*FBXL4*	chr6:993222281	A/C	rs757154231	missense	NM_012160:c.T1739G:p.L580R	0.00001	32
	*UBASH3A*	chr21:43857670	C/T	rs201756769	missense	NM_001001895:c.C1352T:p.T451M	0.0002	30
	*MYH13*	chr17:10206712	T/C	rs767313943	missense	NM_003802:c.A5570G:p.Q1857R	0.00002	27.1
	*FRZB*	chr2:183699592	C/T	rs150679557	missense	NM_001463:c.G962A:p.R321Q	0.00083	24.3
	*TAF1C*	chr16:84213125	G/A	rs61730960	missense	NM_001243158:c.C1036T:p.R346W	0.00041	24.3
	*STRADB*	chr2:202337785	C/G	rs369992593	missense	NM_001206864:c.C301G:p.L101V	0.00008	23.6
	*CLK1*	chr2:201726049	C/A	rs141755850	missense	NM_001162407:c.G428T:p.S143I	0.00041	23
2	*SPTLC3*	chr20:13074186	G/A	rs372930777	missense	NM_018327:c.G788A:p.R263Q	0.00008	35
	*CAPN2*	chr1:223947063	A/G	.	missense	NM_001146068:c.A1175G:p.E392G	0	34
	*COL17A1*	chr10:105807514	C/T	rs757388768	missense	NM_000494:c.G2318A:p.G773E	0.00041	34
	*HCLS1*	chr3:121363691	C/T	rs757006680	missense	NM_001292041:c.G373A:p.G125R	0.00001	34
	*STARD9*	chr15:42930971	C/T	rs369566419	missense	NM_020759:c.C520T:p.R174W	0.0001	33
	*CNST*	chr1:246829203	C/T	rs766380272	missense	NM_152609:c.C2174T:p.S725F	0.00001	28.4
	*STXBP5L*	chr3:120976023	C/T	rs184420053	missense	NM_001308330:c.C1675T:p.L559F	0.00083	26.4
	*DNAH3*	chr16:20975280	G/A	rs376279103	missense	NM_017539:c.C9926T:p.S3309L	0.00008	26
	*TENM4*	chr11:78383268	G/A	rs747100917	missense	NM_001098816:c.C5603T:p.A1868V	0.00001	25.6
	*MYOM3*	chr1:24421405	G/C	rs200854393	missense	NM_152372:c.C866G:p.S289C	0.0004	25.1
	*ASIC2*	chr17:31351024	C/T	rs199589382	missense	NM_001094:c.G1051A:p.A351T	0.0002	23.8
	*NMRK2*	chr19:3933697	T/G	.	splicing	.	0	23.8
	*APOB*	chr2:21252574	C/A	rs778274241	missense	NM_000384:c.G1554T:p.K518N	0.00002	23.4
	*DERL2*	chr17:5384651	C/T	rs202210923	missense	NM_001304777:c.G289A:p.V97I	0.00083	22.9
	*RIC1*	chr9:5762545	G/A	rs771929691	missense	NM_001206557:c.G1886A:p.R629H	0.00041	22.8
	*DLL3*	chr19:39996056	G/A	.	missense	NM_016941:c.G1058A:p.R353K	0	22.4
	*TMEM143*	chr19:48863405	G/A	rs544787964	missense	NM_001303539:c.C293T:p.A98V	0.00083	20.2

Filter 1: Includes only affected carriers; Filter 2 (less stringent): Includes one of the healthy sisters as a carrier. Position is based on reference genome hg19; MMAF: max minor allele frequency; CADD: combined annotation dependent depletion.

**Table 2:** The seventeen variants with MMAF < 0.001, CADD > 20 in Family 2.

Filter	Gene	Position	Ref/Alt	SNP	Type	Change	MMAF	CADD
1	*AKR1B1*	chr7:134136457	C/T	rs201718247	missense	NM_001628:c.G115A:p.G39R	0.00083	34
	*SLC25A25*	chr9:130864666	C/T	rs748220703	missense	NM_001006641:c.C494T:p.T165M	0.0004	34
	*RYR3*	chr15:33893707	C/T	rs760906719	missense	NM_001036:c.C1876T:p.R626W	0.00041	34
	*RUFY1*	chr5:178987155	A/T	rs754852607	missense	NM_001040451:c.A116T:p.Q39L	0.00008	28.6
	*TIPIN*	chr15:66641436	T/C	rs200514985	missense	NM_001289986:c.A134G:p.D45G	0.00041	27.9
	*C1orf228*	chr17:45166747	C/T	rs575641425	missense	NM_001145636:c.C595T:p.P199S	0.00041	26.3
	*HECTD4*	chr12:112677734	T/C	rs779868916	missense	NM_001109662:c.A4654G:p.I1552V	0.00002	25.2
	*BCL6*	chr3:187444624	T/C	rs747910667	missense	NM_001130845:c.A1603G:p.R535G	0.0001	23.4
	*CA12*	chr15:63618533	C/T	rs149256486	missense	NM_001293642:c.G803A:p.G268E	0.0002	23.3
2	*UTP11L*	chr1:38489295	C/T	rs771377582	missense	NM_016037:c.C757T:p.R253C	0.0002	35
	*PAX7*	chr1:18961015	G/A	rs369607271	missense	NM_001135254:c.G304A:p.G102S	0.00008	34
	*FNDC3B*	chr3:171969145	C/T	rs190147254	missense	NM_001135095:c.C604T:p.R202C	0.00083	31
	*ZMYM4*	chr1:35836075	G/A	.	missense	NM_005095:c.G1028A:p.G343D	0	24.6
	*WDFY4*	chr10:50013303	A/G	rs748753983	splicing	.	0.00005	23.7
	*NSD2*	chr4:1957024	C/G	rs748922675	missense	NM_001042424:c.C2475G:p.H825Q	0.00041	23.6
	*MED14*	chrX:40552004	G/A	rs763899660	missense	NM_004229:c.C1801T:p.R601C	0.00026	22.7
	*C17orf53*	chr17:42225596	G/A	rs377372267	missense	NM_001171251:c.G425A:p.S142N	0.0004	21.8

Filter 1: Includes only carriers affected with breast and lung cancer; Filter 2 (less stringent): All carriers affected with breast cancer (including those with also lung cancer). Position is based on reference genome hg19; MMAF: max minor allele frequency; CADD: combined annotation dependent depletion.

**Table 3:** The thirty-five variants with MMAF < 0.001, CADD > 20 in Family 3.

Filter	Gene	Position	Ref/Alt	SNP	Type	Change	MMAF	CADD
1	*PRKD1*	chr14:30108088	A/C	.	missense	NM_001330069.2:c.743T > G:p.F248C	0	22.3
2	*TNRC6C*	chr17:76094616	C/T	rs367710467	missense	NM_001142640.1:c.4607C > T:p.S1536L	0	33
	*STAB1*	chr3:52554136	G/C	.	missense	NM_015136.3:c.5412G > C:p.E1804D	0	33
	*THOP1*	chr19:2799789	G/A	.	missense	NM_003249.5:c.589G > A:p.G197R	0	33
	*SPDYA*	chr2:29072798	TG/T	.	splicing	NM_001142634.2:c.934del:p.E312KfsTer20	0	32
	*TMX2-CTNND1*	chr11:57505465	C/T	rs375390370	missense	NM_001347890.1:c.331C > T:p.R111C	0	32
	*ZNF793*	chr19:38023257	G/A	rs200503532	splicing	NM_001013659.3:c.16-1G > A	0	32
	*NOMO3*	chr16:16363998	C/T	.	missense	NM_001004067.4:c.1915C > T:p.R639C	0.00017	32
	*USP17L10*	chr4:9213003	T/A	.	nonsense	NM_001256852.1:c.621T > A:p.C207Ter	0	32
	*CNR2*	chr1:24201377	G/A	rs201829495	missense	NM_001841.3:c.731C > T:p.A244V	0	28.2
	*ZNF507*	chr19:32845840	T/C	.	missense	NM_001136156.2:c.2104T > C:p.C702R	0	27.2
	*USP40*	chr2:234399901	G/GA	.	splicing	NM_001365479.1:c.2923dup:p.S975FfsTer65	0	27
	*TUBGCP6*	chr22:50656236	G/A	rs138609686	missense	NM_020461.4:c.5389C > T:p.R1797C	0.0000649	26.6
	*AGAP3*	chr7:150839000	T/G	.	missense	NM_001281300.2:c.827T > G:p.F276C	0	25.9
	*ITPR1*	chr3:4706906	G/A	.	missense	NM_001099952.3:c.1639G > A:p.A547T	0	25.8
	*SPDL1*	chr5:169028384	AG/A	.	splicing	NM_001329639.2:c.1426del:p.E476KfsTer19	0	25.7
	*RUNX1*	chr21:36164605	A/C	.	missense	NM_001001890.3:c.1189T > G:p.S397A	0	25.5
	*TCF3*	chr19:1646413	G/C	.	missense	NM_001136139.4:c.86C > G:p.P29R	0	25.2
	*NT5C1B-RDH14*	chr2:18745234	C/T	rs147855687	missense	NM_001002006.3:c.1661G > A:p.R554H	0.000454	25
	*ABHD6*	chr3:58279442	G/A	rs144907290	missense	NM_001320126.2:c.964G > A:p.D322N	0	24.9
	*EPHA2*	chr1:16456073	C/T	.	missense	NM_001329090.2:c.2519G > A:p.R840Q	0	24.8
	*HSPA6*	chr1:161494581	G/C	.	missense	NM_002155.5:c.133G > C:p.A45P	0	24.4
	*MYO16*	chr13:109507831	C/T	.	missense	NM_001198950.3:c.1289C > T:p.T430M	0	23.8
	*SMC4*	chr3:160135704	T/C	rs41272953	missense	NM_001002800.3:c.1631T > C:p.I544T	0.000115	23.5
	*PRKAR2A*	chr3:48884770	T/G	.	missense	NM_001321982.2:c.260A > C:p.E87A	0	23.5
	*USP17L22*	chr4:9270417	G/GA	.	splicing	NM_001256863.1:c.1073_1074insA:p.S358RfsTer20	0	23.3
	*ZYX*	chr7:143079991	G/T	rs150223874	missense	NM_001010972.2:c.599G > T:p.W200L	0	23.1
	*ITFG1*	chr16:47494745	T/G	rs376408976	splicing	NM_001305002.1:c.−132 + 281A > C	0	23.1
	*NYNRIN*	chr14:24883816	T/C	.	missense	NM_025081.3:c.2861T > C:p.I954T	0	22.6
	*NIM1K*	chr5:43280642	C/G	.	missense	NM_153361.4:c.1122C > G:p.N374K	0	22.3
	*DICER1*	chr14:95570153	T/C	.	missense	NM_001271282.3:c.3580A > G:p.R1194G	0	21.8
	*NXNL1*	chr19:17571431	G/A	rs377352923	missense	NM_138454.2:c.248C > T:p.T83M	0	21.5
	*TRPV6*	chr7:142574988	G/T	rs139115329	missense	NM_018646.6:c.514C > A:p.L172M	0	21.2
	*ZNF320*	chr19:53384145	G/C	.	missense	NM_001351773.1:c.1234C > G:p.L412V	0	21
	*ZNF320*	chr19:53384768	A/G	rs144964547	missense	NM_001351773.1:c.611T > C:p.L204P	0.000907	20.7

Filter 1: Includes only affected carriers; Filter 2 (less stringent), Includes that one of the healthy parents could be a carrier. Position is based on reference genome hg19; MMAF: max minor allele frequency; CADD: combined annotation dependent depletion.

## Data Availability

Data available in article supplementary files.

## Supplementary Materials

The following supporting information can be downloaded at: https://www.mdpi.com/article/10.3390/biomedicines10051004/s1, Table S1: Clinical characteristics of all patients in the study. Table S2: Five variants in 64 hereditary cancer-related genes with MMAF < 0.2 and one SNP in the study. Table S3: All exonic variants found in the WES with MMAF < 0.001 in all families. Table S4: Western blot analysis of MYH13, UTP11L and PAX7 identified as candidates in Family 1 and Family 2. Figure S1: Ectopic expression of MYH13 identified as a candidate gene in Family 1 and its biological effects analyzed by KEGG pathway analysis. Figure S2: Ectopic expression of UTP11L identified as a candidate gene in Family 2 and its biological effects analyzed by KEGG pathway database.
