# Supplementary material for: Whole-Exome Sequencing of Germline Variants in Non-BRCA Families with Hereditary Breast Cancer
Source: Biomedicines. 2022 Apr 26;10(5):1004. doi: 10.3390/biomedicines10051004 (PMC9138793; doi:10.3390/biomedicines10051004)
Supplement: Supplementary file 1 [file biomedicines-10-01004-s001.zip › supplementary/SuppleFigures.pptx]

## Slide 1
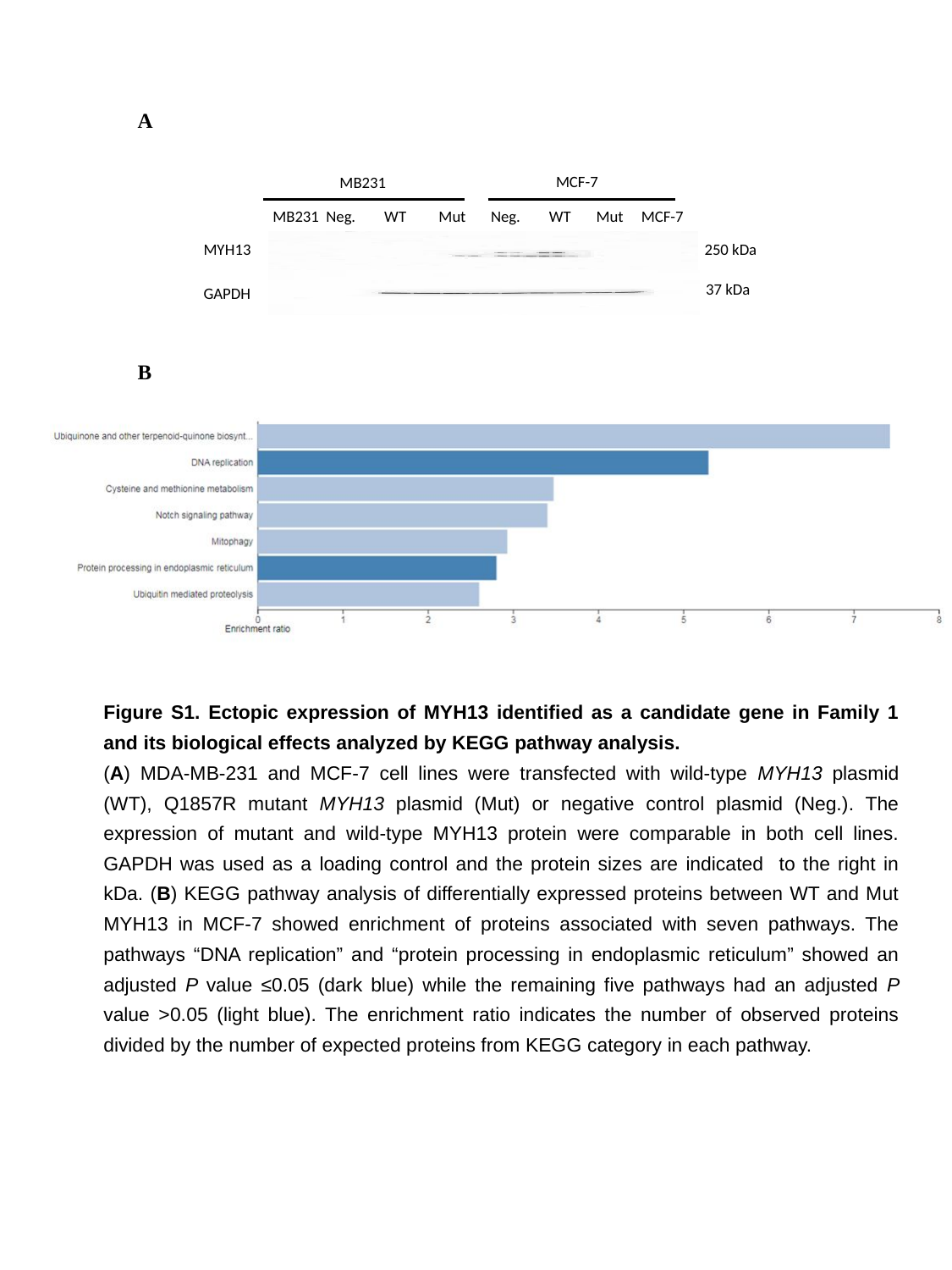

A
 MCF-7
MB231
 MB231 Neg. WT Mut Neg. WT Mut MCF-7
MYH13
250 kDa
37 kDa
GAPDH
B
Figure S1. Ectopic expression of MYH13 identified as a candidate gene in Family 1 and its biological effects analyzed by KEGG pathway analysis.
(A) MDA-MB-231 and MCF-7 cell lines were transfected with wild-type MYH13 plasmid (WT), Q1857R mutant MYH13 plasmid (Mut) or negative control plasmid (Neg.). The expression of mutant and wild-type MYH13 protein were comparable in both cell lines. GAPDH was used as a loading control and the protein sizes are indicated to the right in kDa. (B) KEGG pathway analysis of differentially expressed proteins between WT and Mut MYH13 in MCF-7 showed enrichment of proteins associated with seven pathways. The pathways “DNA replication” and “protein processing in endoplasmic reticulum” showed an adjusted P value ≤0.05 (dark blue) while the remaining five pathways had an adjusted P value >0.05 (light blue). The enrichment ratio indicates the number of observed proteins divided by the number of expected proteins from KEGG category in each pathway.

## Slide 2
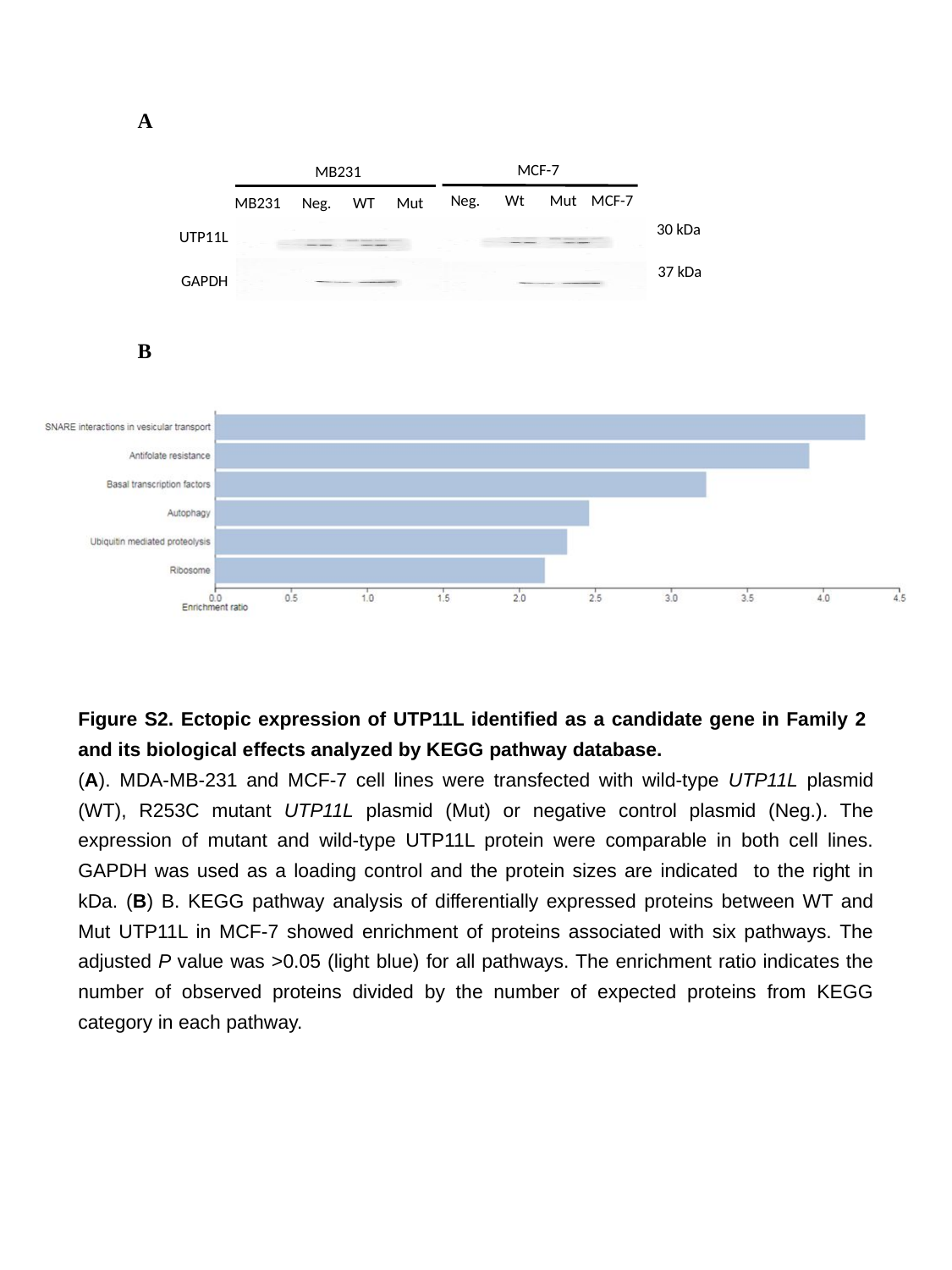

A
 MCF-7
Neg. Wt Mut MCF-7
MB231
MB231 Neg. WT Mut
30 kDa
UTP11L
37 kDa
GAPDH
B
Figure S2. Ectopic expression of UTP11L identified as a candidate gene in Family 2 and its biological effects analyzed by KEGG pathway database.
(A). MDA-MB-231 and MCF-7 cell lines were transfected with wild-type UTP11L plasmid (WT), R253C mutant UTP11L plasmid (Mut) or negative control plasmid (Neg.). The expression of mutant and wild-type UTP11L protein were comparable in both cell lines. GAPDH was used as a loading control and the protein sizes are indicated to the right in kDa. (B) B. KEGG pathway analysis of differentially expressed proteins between WT and Mut UTP11L in MCF-7 showed enrichment of proteins associated with six pathways. The adjusted P value was >0.05 (light blue) for all pathways. The enrichment ratio indicates the number of observed proteins divided by the number of expected proteins from KEGG category in each pathway.
